# Supplementary material for: Evolution of a Multiple Sex-Chromosome System by Three-Sequential Translocations among Potential Sex-Chromosomes in the Taiwanese Frog Odorrana swinhoana
Source: Cells. 2021 Mar 16;10(3):661. doi: 10.3390/cells10030661 (PMC8002213; doi:10.3390/cells10030661)
Supplement: Supplementary file 1 [file cells-10-00661-s001.zip › Supplemental/Figures S.pptx]

## Slide 1
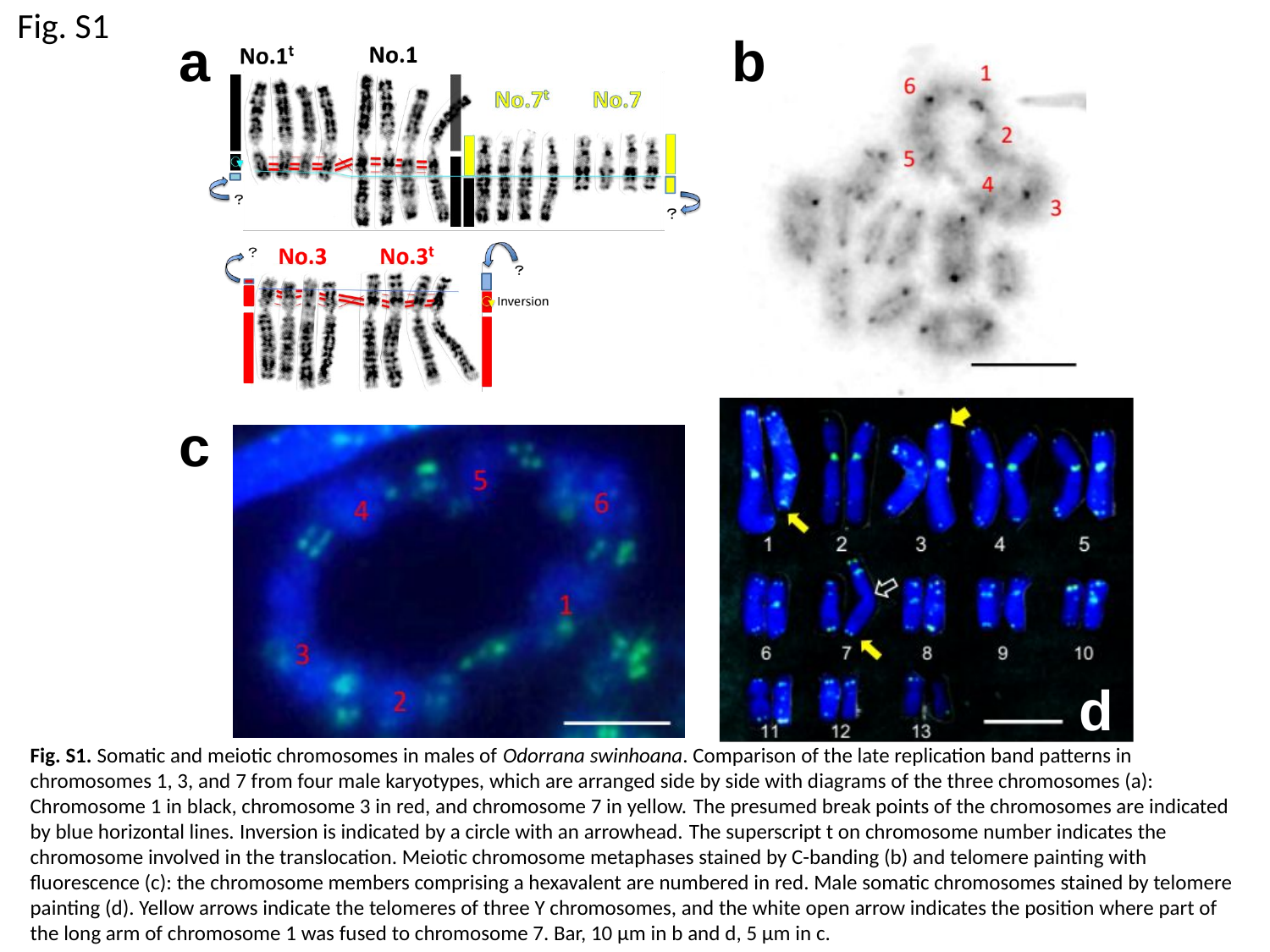

Fig. S1
a
b
c
d
Fig. S1. Somatic and meiotic chromosomes in males of Odorrana swinhoana. Comparison of the late replication band patterns in chromosomes 1, 3, and 7 from four male karyotypes, which are arranged side by side with diagrams of the three chromosomes (a): Chromosome 1 in black, chromosome 3 in red, and chromosome 7 in yellow. The presumed break points of the chromosomes are indicated by blue horizontal lines. Inversion is indicated by a circle with an arrowhead. The superscript t on chromosome number indicates the chromosome involved in the translocation. Meiotic chromosome metaphases stained by C-banding (b) and telomere painting with fluorescence (c): the chromosome members comprising a hexavalent are numbered in red. Male somatic chromosomes stained by telomere painting (d). Yellow arrows indicate the telomeres of three Y chromosomes, and the white open arrow indicates the position where part of the long arm of chromosome 1 was fused to chromosome 7. Bar, 10 μm in b and d, 5 μm in c.

## Slide 2
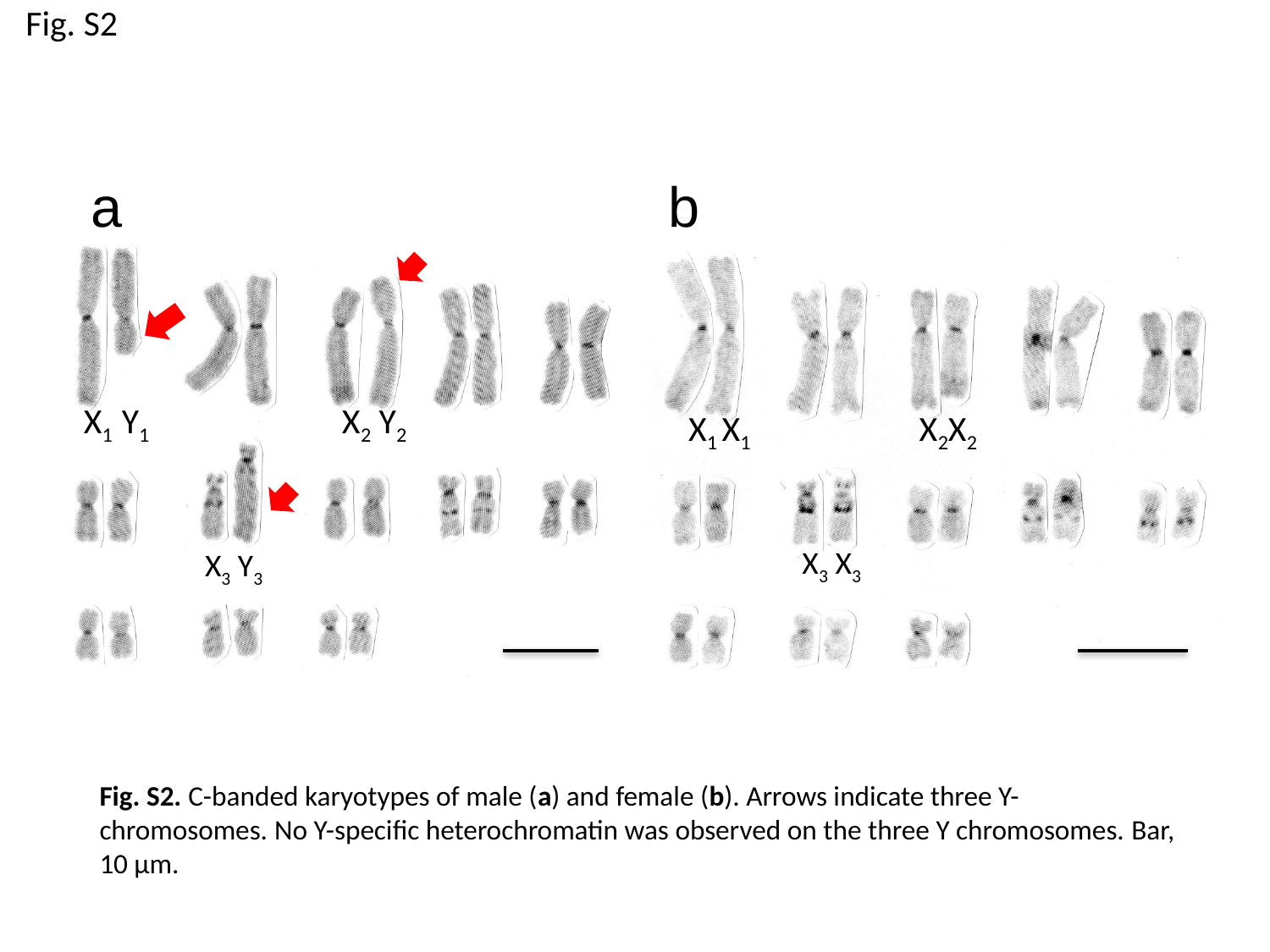

Fig. S2
a
b
X1 Y1 X2 Y2
X1 X1 X2X2
X3 X3
X3 Y3
Fig. S2. C-banded karyotypes of male (a) and female (b). Arrows indicate three Y-chromosomes. No Y-specific heterochromatin was observed on the three Y chromosomes. Bar, 10 μm.

## Slide 3
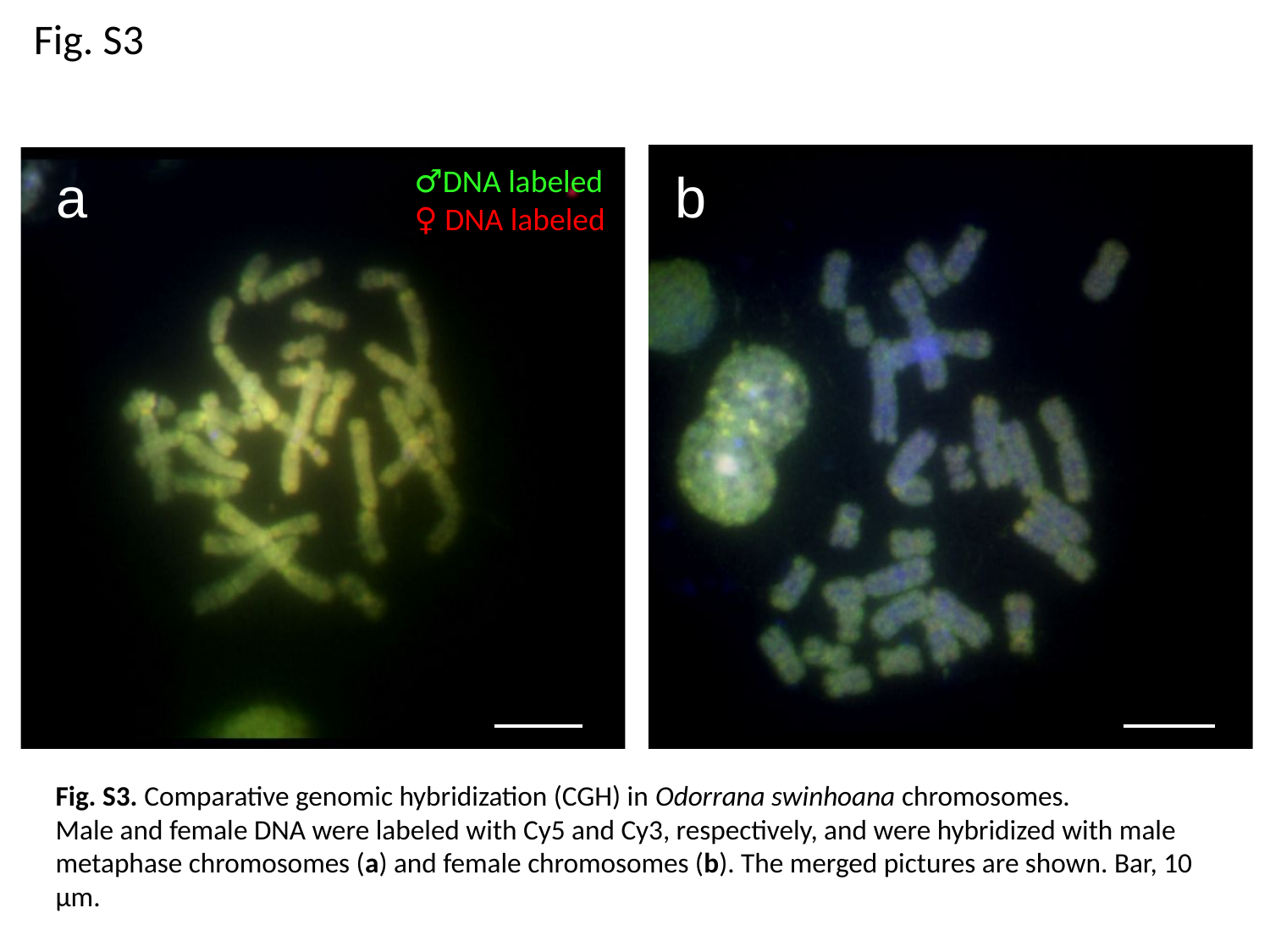

Fig. S3
a
♂DNA labeled
♀ DNA labeled
b
Fig. S3. Comparative genomic hybridization (CGH) in Odorrana swinhoana chromosomes.
Male and female DNA were labeled with Cy5 and Cy3, respectively, and were hybridized with male metaphase chromosomes (a) and female chromosomes (b). The merged pictures are shown. Bar, 10 μm.

## Slide 4
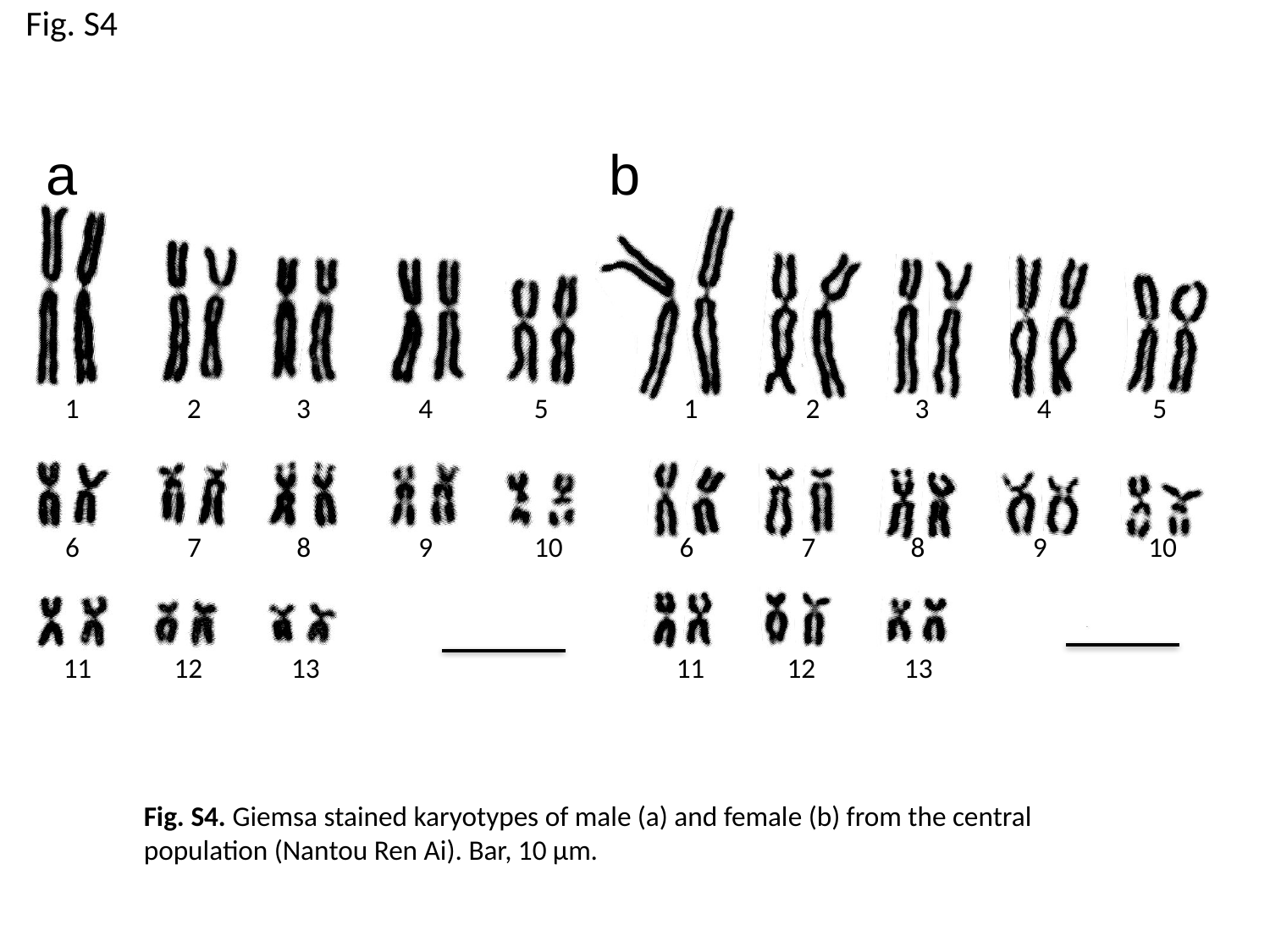

Fig. S4
a
b
1 2 3 4 5
1 2 3 4 5
6 7 8 9 10
6 7 8 9 10
11 12 13
11 12 13
Fig. S4. Giemsa stained karyotypes of male (a) and female (b) from the central population (Nantou Ren Ai). Bar, 10 μm.

## Slide 5
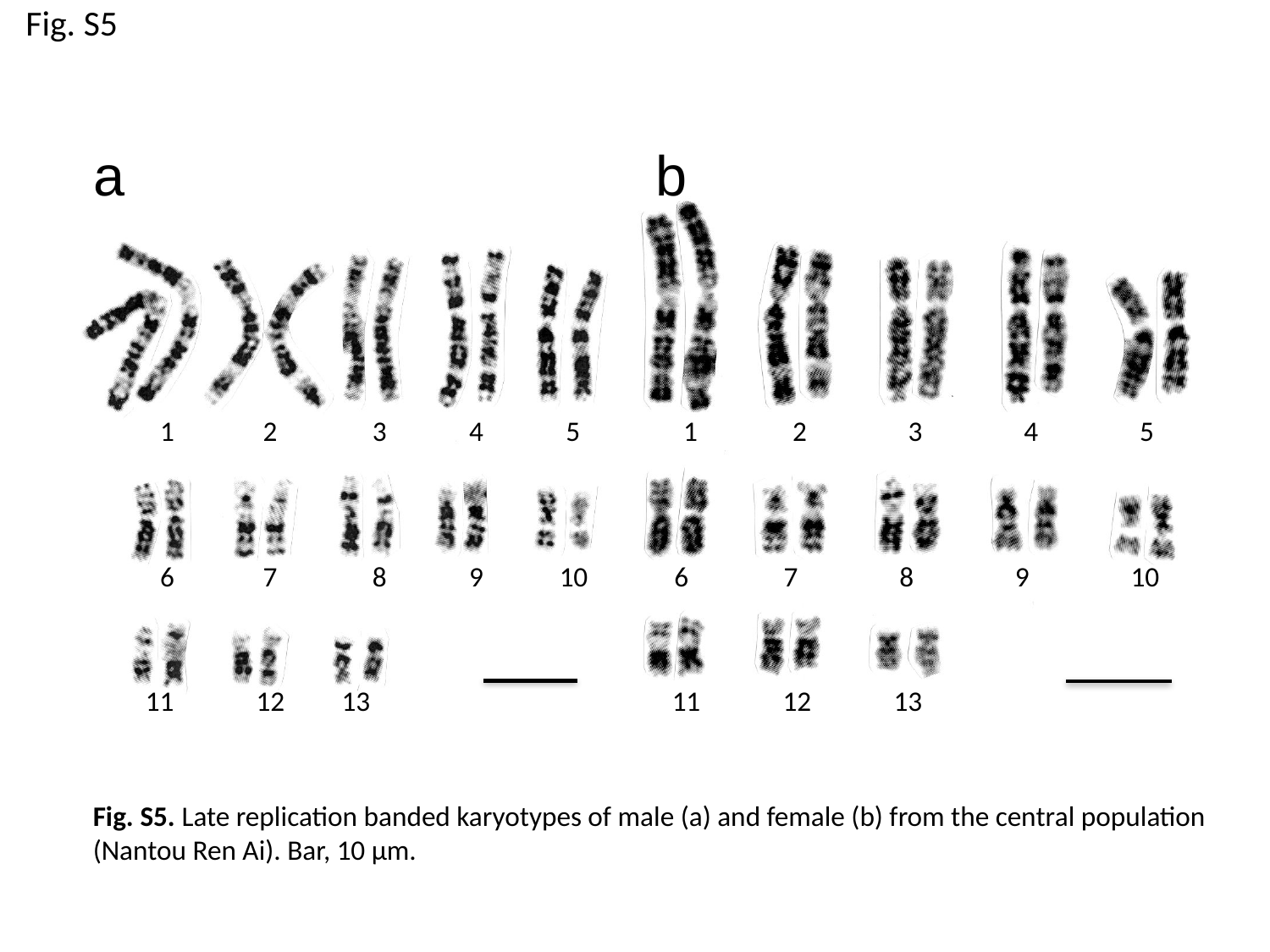

Fig. S5
a
b
1 2 3 4 5
1 2 3 4 5
6 7 8 9 10
6 7 8 9 10
11 12 13
11 12 13
Fig. S5. Late replication banded karyotypes of male (a) and female (b) from the central population (Nantou Ren Ai). Bar, 10 μm.

## Slide 6
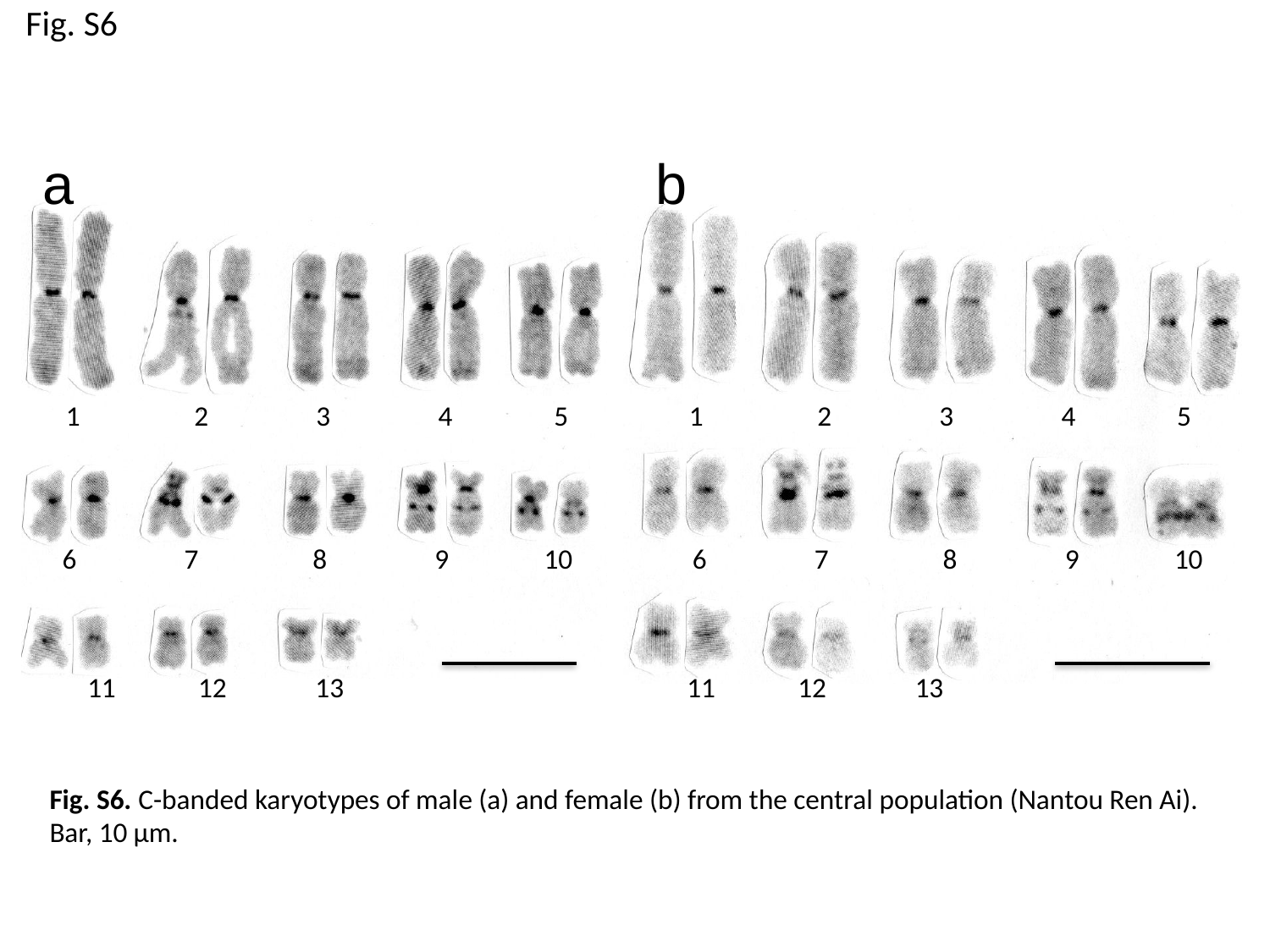

Fig. S6
a
b
1 2 3 4 5
1 2 3 4 5
6 7 8 9 10
6 7 8 9 10
11 12 13
11 12 13
Fig. S6. C-banded karyotypes of male (a) and female (b) from the central population (Nantou Ren Ai). Bar, 10 μm.
