# Supplementary material for: Evolution of a Multiple Sex-Chromosome System by Three-Sequential Translocations among Potential Sex-Chromosomes in the Taiwanese Frog Odorrana swinhoana
Source: Cells. 2021 Mar 16;10(3):661. doi: 10.3390/cells10030661 (PMC8002213; doi:10.3390/cells10030661)
Supplement: Supplementary file 1 [file cells-10-00661-s001.zip › Supplemental/Table S1.docx]

**Table S1.** Collecting stations of *Odorrana swinhoana* and number of frogs examined

| Population | City | Latitude | Altitude | Date | No. of frogs | Sex | Hetero^1)^ | Homo |
| --- | --- | --- | --- | --- | --- | --- | --- | --- |
| Northern 1 | New | 24.948419 | 121.578521 | 2018, | 4 | Male | 4 | 0 |
|  | Taipei |  |  | 2019 | 3 | Female | 0 | 3 |
| Northern 1 | New | 24.948419 | 121.578521 | 2019 | 5 | Male | 5 | 0 |
| (F1)^2)^ | Taipei |  |  |  | 5 | Female | 0 | 5 |
| Northern 2 | New | 24.890256 | 121.567175 | 2019 | 2 | Male | 0 | 2 |
|  | Taipei |  |  |  | 2 | Female | 0 | 2 |
| Central | Nantou | 24.074926 | 120.994927 | 2018 | 3 | Male | 0 | 3 |
|  | Ren Ai |  |  |  | 3 | Female | 0 | 3 |

1) Three heteromorphic chromosomes.

2) Offspring from a mating between the male and female from Northern 1 population.
